# An exploration of community food pantries in Scotland: strategic and operational perspectives on addressing food insecurity and health inequalities

**DOI:** 10.1186/s12889-024-20421-z

**Published:** 2024-10-18

**Authors:** Anna Baillie, Kathryn Skivington, Gillian Fergie

**Affiliations:** MRC /CSO Social and Public Health Science Research Unit, School of Health and Wellbeing, University of Glasgow, 90 Byres Road, Glasgow, G12 8TB UK

**Keywords:** Community level, Health inequalities, Pantry, Food insecurity, Scotland

## Abstract

**Introduction:**

: Food insecurity and health are inextricably linked. Since 2008, Scotland has witnessed a proliferation of both food insecurity and emergency food provision. There is a recognised commitment from Scottish Government to ‘end the need for food banks’, however, the food aid landscape was ‘turbo-charged’ during COVID-19 leading to intense expansion and diversification of food-based projects, including the development of *community food pantries (CFPs)*. These ‘new’ models are relatively under-researched, meaning we do not adequately understand their potential or realised impacts on food insecurity and health. This study aims to fill that gap.

**Methods:**

A qualitative methodology was used to collect and analyse data from in-depth interviews with 10 representatives from both operational and policy settings related to food insecurity in Scotland. In addition, we conducted an analysis of policy documentation from Scottish Government related to tackling food insecurity to understand how CFPs fit into its overall strategy to transition away from food bank use.

**Results:**

We found there were variations in conceptualisations of CFPs and how they operate, challenges related to addressing food insecurity at a community level and varied narratives around the role of community level interventions in tackling health inequalities. Choice and access to services were viewed as central components to the pantry model. However, there were significant challenges faced by CFPs, including territorialism, funding and food supply. Articulations of health were often multi-layered and complicated with strong recognition of the social determinants as well as acknowledgement of the limitations of tackling food insecurity and health inequalities solely at the community level.

**Conclusions:**

Despite a commitment to transition away from emergency food provision, CFPs in Scotland appear to face many of the same issues as food banks, particularly those which impact health. Urgent critique of their reliance on surplus food redistribution is required alongside investigation of how these ‘new’ models are experienced by the people who access them. Further expansion of these models should be viewed with caution and in the same vein as traditional emergency food provision: a symptom of, rather than a solution to, the problem of food insecurity.

**Supplementary Information:**

The online version contains supplementary material available at 10.1186/s12889-024-20421-z.

## Introduction

Food insecurity is a complex policy problem, which high income countries internationally are failing to tackle comprehensively [1–3]. Broadly understood as ‘the inability to acquire or eat an adequate quality or sufficient quantity of food in socially acceptable ways (or the uncertainty of being able to do so)’ [4], food insecurity has proliferated across Europe since the 2008 financial crash, and particularly so in the UK, which, along with Ireland, has witnessed some of the sharpest increases in households who experience it [5, 6]. This is of deep concern to researchers and policymakers in the realms of health inequalities, who have long viewed increasing levels of food insecurity as a deepening public health crisis [2, 7–11]. That food is crucial to health is well established and undisputed [6, 12]; repeated studies have linked experiences of food insecurity with a panoply of physical health conditions [2, 6, 12, 13], including ‘obesity, malnutrition, hypertension, iron deficiency and impaired liver function’ [14], demonstrating a causal relationship between the inability to afford a nutrient-dense diet and nutritional health and wellbeing [6, 8, 13]. However, the full impact of food insecurity on health extends far beyond nutrition. A recent meta-ethnography revealed a complex matrix of potential pathways and mechanisms through which food insecurity impacts on health [12]. These span across a wide spectrum, including biological impacts and the exacerbation of existing health issues [6, 14], pervasive psychosocial harms [12–19], and related mental health impacts [9, 11, 15–17, 19]. These consequences for health accumulate and coalesce to produce the ‘hidden cost’ burdens of food insecurity [13], which people experiencing it have to navigate and cope with, ultimately producing the what Bambra et al. term the ‘everyday realities of health inequalities’ [6].

Described as ‘foodbank Britain’ [16] by some, the UK has witnessed a proliferation of food assistance programmes and emergency food providers over the past two decades [3, 9]. Broadly speaking, in the UK, ‘food banks are places where people can go to receive free groceries, usually just enough for a couple of days and that can be carried home’ [9]. Food banks, and other food-based responses, which offer free or low cost food for people experiencing food insecurity, often find themselves at the heart of debates about policy action [20]. Indeed, the vast majority of research into experiences of food insecurity takes place via the vector of food banking, with questions raised, in both academic and policy spaces in the UK, about whether they solve or exacerbate the problem of food insecurity [3], given their inability to address its root causes of poverty and income crisis [1, 15, 17] as well as their limited capacity to respond to the scale of need [21]. This is further compounded by well-documented concerns about the negative consequences for nutritional health, such as the inability to access appropriate and varied diets [9] and general wellbeing, such as psychosocial harms [12, 15, 17, 19], for people who use them.

Despite this, the COVID-19 pandemic ‘turbo-charged’ [3] the prevalence of food-based responses across the UK, and as food insecurity levels have risen [22, 23], so too has the number of food banks and food aid providers. This expansion has also led to diversification. Owing to the long running and sustained critique on their modes of operation [9, 10, 20, 24], food banks have sought to rebrand their models and change their ways of working [9]. Now there are increasing numbers of initiatives which align with Loopstra’s definition of *community food programmes* [1], which are seen as an evolution and enhancement of the food bank model through:

*additional services such as benefits counselling with a caseworker*,* debt counselling*,* cooking classes or fuel vouchers’ or through a ‘community shop*,* which through a membership structure (possibly operated through volunteering time or a low membership fee)*,* members make their own choices from the donated or surplus foodstuffs available* [1].

These seemingly new models [25], often badged as ‘more than food’ [9, 12, 26] are emerging across Scotland [27], with *community food pantries (CFPs)* in particular, becoming more common in recent years. The Scottish Pantry Network, established in 2018, defines pantries as supports that ‘can be accessed in local communities where, for a small membership fee, heavily discounted food is available’ [28]. Their increasing presence in communities in Scotland comes alongside an explicit policy focus on drivers and solutions of food insecurity [29], and commitment from Scottish Government to ‘end the need for food banks’ [30]. Such *community food pantries (CFPs)* have received relatively less research attention meaning it is unclear how they operate and, as such, whether they **address**,** or potentially reproduce**, existing limitations in emergency food provision or generate new/alternative concerns. Given the demonstrable importance of food security for health, it is important to understand how these models and projects are understood and function within communities. The majority of research in the UK uses food banks as a basis for understanding experiences of food insecurity, whilst there is scant research into this potentially new phenomenon of food provision. This study aims to bridge this gap and increase our understanding of both how CFPs are conceptualised and their role in tackling food insecurity and health inequalities at a community level.

## Study aim

The goal of this research was to explore how stakeholders involved in the development of CFPs, or similar projects, frame potential and realised impacts of these initiatives on individuals, organisations and communities with a particular focus on how health is framed, and how the social determinants of health feature.

## Study design

This was a qualitative study using an interpretivist / social constructivist approach [31, 32]. We aimed to build a picture of CFPs and their perceived role in tackling food insecurity and health inequalities by drawing on relevant policy documentation and the perspectives of a range of individuals involved in both frontline and policy settings related to food insecurity in Scotland. We conducted ten semi-structured interviews to explore descriptions of CFPs, their impacts and articulations of health. A topic guide for the interviews was developed specifically for this study and is available as a supplementary file alongside this paper (see Additional file 1). Our recruitment was purposive and focussed on Glasgow, where there has been a flurry of development of pantry models of food provision [33]. KS and GF along with the support of a research assistant (SP, please see acknowledgements) conducted the interviews, all of which took place online (January-April 2022), for around 1 h and were recorded and transcribed using MS Teams and processed using N-Vivo software. Table 1 below details the range of perspectives included.

**Table 1:** Interview participants

Participant organisation / role	No.
CFPs	4
Traditional emergency food provision, i.e., food banks	2
Food policy experts	3
Community food development worker	1

In order to understand the backdrop to individuals’ accounts, we also reviewed relevant Scottish policy documentation. Scottish Government’s ‘food insecurity’ web page was searched for references and links to relevant documents, i.e. those related to the response to food insecurity. We included documents published after 2016, for reasons mentioned above. The documents included were:

i) Dignity: Ending hunger together in Scotland, 2016 [29]: The report produced by the independent working group on food poverty which set out a number of recommendations to transition away from typical emergency food provision.
ii) Review of the Fair Food Transformation Fund, 2018 [34] : In response to the Dignity: Ending hunger together in Scotland report, the Scottish Government set up a fund to ‘support projects that give a more dignified response to food poverty and help move away from emergency food aid as the main response’, this report reviews the funded projects.
iii) Food insecurity and poverty: United Nations, Scottish Government Response, 2021 [35] : details Scottish Government’s response to a joint letter from the food and poverty UN Special Rapporteurs. It details ‘Scotland’s human rights approaches to the challenges of food insecurity and poverty’ [35].
iv) Food Insecurity Fact Sheet, 2022 [36]: This briefing provides information for applicants to the Investing in Communities Fund (running from 2023 to 2026), whose goal is to respond to food insecurity. It details the core principles which Scottish Government are looking to fund as well as detailing a CFP case study.

These documents provide a helpful chronology of the evolving policy context of food insecurity in Scotland and where novel approaches, such as CFPs, fit into its overall strategy to ‘end the need for foodbanks’.

### Analysis

We used thematic analysis [37] to triangulate the data from both semi-structured interviews and the policy documents. An abductive coding framework was developed according to the aims of the study and applied across the data set. This framework consisted of deductive codes related to the study aims, for example ‘modes of operation’; ‘role of CFPs’ and ‘articulations of health’ as well as inductive codes which were developed in response to the research teams’ engagement with the data, for example ‘dignity’; ‘competition between projects’ and ‘supply issues’. We took this abductive approach (i.e., combining both deductive and inductive coding) as it allowed flexibility in our interpretation of the data and a deeper understanding of the identified themes. This is a common approach to data analysis in interpretative qualitative studies [31]. AB uploaded each interview transcript and policy document to N-Vivo and conducted the first round of coding, KS and GF then cross-coded. The research team met regularly to discuss the identified themes and agree our shared interpretation of the data according to the study aims.

## Results

Analysis of interview accounts and policy documents suggests a variety of narratives around the role of CFPs and their potential or realised impacts for the people who use them, both in terms of their capacity to tackle food insecurity as well as how they respond to wider health needs and issues of communities. Analytical themes are grouped into three categories: *Conceptualisations of community food pantries*, *Challenges of addressing food insecurity at a community level* and *Articulations of health inequalities in relation to food insecurity & CFPs*. Each category is discussed in turn in the following sections.

### Conceptualisations of community food pantries

Overall, there were differing narratives of what a CFP is and what it sets out to do, particularly in relation to the role they play in responding to food insecurity. Despite direct references to health or health inequalities being relatively absent from participant and policy conceptualisations, the pervasive psychosocial harms resulting from food insecurity were widely acknowledged with choice and dignity emerging as core concepts in the descriptions of CFPs. Access to wider services was often described as the key mechanism through which CFPs might resolve experiences of food insecurity (beyond the provision of food) however *which* services and *how* the pantry facilitates access appeared unclear. These points will now be discussed in turn.

#### How community food pantries operate

Participants described CFPs in common ways, where their primary function was provision of food rather than initiatives designed to tackle health inequalities directly. Most described CFPs as projects which were open once or twice a week, and tended to follow a membership model e.g., members paid £2.50, to have access to a higher value of food, e.g., £10–15 worth, which was almost always sourced from a waste-food redistribution charity. However, there were differences in reports of who used the pantries, and ultimately different views on who the pantries were intended to be used by, or even to whom they were available. Some participants described pantries as destigmatising in nature because they were intended to be open *‘for everyone’*, with no requirement to *‘evidence how skint’* users are, akin to a shop, as stated by one of the pantry representatives:

*with pantries there’s a lot of differences because it feels like you’re just going on a shop*,* you know*,* going in a supermarket and the stigma … could be lessened*** CFP representative 1**.

However, others described ‘screening’ and ‘eligibility criteria’, inferring that this open-door policy was not always in place, and this was justified as necessary in order to have sufficient food to reach those who need it most.

#### Pantries are different / better than food banks

Across accounts in interviews and policy documents, CFPs were routinely conceptualised in terms of how they differ, or could potentially differ, from traditional food banks. Notably, this preference for pantries over food banks was often related to the concept of *dignity* rather than necessarily the pantries’ capacity to adequately resolve experiences of food insecurity, for example via removing or significantly mitigating experiences of hunger, worrying about food, skipping meals and /or knowingly reducing the quality and quantity of food being consumed [38]. This is well demonstrated in the Dignity: Ending hunger together in Scotland Report, which positions ‘dignity’ front and centre to food provision:

*Any organisation funded to tackle food poverty through the Scottish Government must demonstrate how its approach promotes dignity and*,* for emergency providers*,* how they seek to make the transition from emergency response to dignified provision integrated within wider community settings* [29].

One key aspect of divergence between food banks and CFPs is the apparent *choice* provided by these models. In the context of pantries this was largely conceptualised in the way in which the pantry functions, i.e., members can walk around and choose items themselves.

*They don’t feel empowered [at a food bank] because the food that is being provided to them [is] already prepackaged**** CFP representative 1***.

Choice appears to be deeply entwined with understandings of what a ‘dignified’ response is for participants. This theme is strongly threaded throughout the policy documents, indeed one of the four ‘dignity principles’ originally developed by the independent working group on food poverty in 2016 to guide community level responses to food insecurity is ‘[l]eave people with the power to choose’ [29].

The potential to enable users more choice and, as such dignity, are therefore central to discursive constructions of pantries across accounts, and what, for some, particularly those involved in the delivery of CFPs, set the model apart from food banks.

#### CFPs provide crisis support?

Whether pantries exist for people experiencing crisis, short-, or long-term support also varied, including amongst pantry providers themselves. Some participants clearly located pantries within the emergency food aid landscape:

*It is a definite emergency response. We get loads of crisis referrals every week.**** CFP representative 3***.

Whilst others viewed the pantry model as offering consistent regular support beyond the typical short-term crisis response provided by food banks. Despite all participants describing pantries as largely following a membership model, implying a longer-term relationship with their beneficiaries, some conceptualisations appeared to encourage short-term or time-limited use:

*I think this is where you’re in crisis*,* absolutely*,* we’ll help you get out of crisis*,* but then we move you on.**** CFP representative 2***.

In addition, some participants questioned CFPs’ suitability for providing crisis support to people experiencing an income crisis thanks to the perceived cost of access (via the membership fee) and very limited opening times. How pantries operate as part of emergency responses then remains unclear - while there was some agreement on the model potentially enabling dignity for users, accounts suggest the ongoing need for crisis support might not be fully met.

#### Beyond food: access to wider services

Access to wider services was fervently considered to be integral to CFPs, again in a way that set them apart from food banks, as one pantry representative details:

*It was never about just the food*,* it was always about the wrap around services…upskilling people to help them back into employment*,* welfare rights*,* mental health services*,* all those types of services in the one place where people can access them**** CFP representative 2***.

It was these wider aspects of the initiatives that appeared to participants as the main contributions to addressing food insecurity beyond provision of food. This is reflected strongly throughout the interview data and is also evident in policy documents, all of which advocate for a shift away from ‘stand-alone emergency food provision’ towards ‘community-food’ hub models [29], which facilitate access to wider services [29, 34–36].

Despite this strong consensus that food projects should be linked to wider support services, there was relative ambiguity about what these services were and how people accessed them, particularly in the context of CFPs and novel approaches to food provision. Across the more recent policy documents analysed, i.e., the Scottish Government response to the UN Rapporteur on food poverty and the Food Insecurity Fact Sheet [35, 36], there are clear efforts to frame Scottish Government’s primary approach to tackling food insecurity as one which prioritises income maximisation, or ‘cash-first’ responses, with facilitating access to ‘money advice’ cited as integral, particularly in the context of food based projects [36]. ‘Cash-first’ is a term gaining increasing traction in Scotland and the UK in the context of food insecurity responses [39]. This approach consists of prioritising supports, such as welfare rights advice or access to discretionary funds, which can provide people experiencing food insecurity with money to spend rather than access to emergency food provision. Whilst four participants cited the concept of ‘cash-first’ responses to food insecurity and wider work to develop ‘robust referral pathways’ between services, this was rarely in relation to CFPs directly. The potential services available at pantries appeared to range across a wide matrix of support systems from befriending, social groups and skills-based classes to statutory family support and health services, with often unclear articulations of *how* a particular pantry facilitated access to these wider supports or how some of the wider supports addressed or mitigated experiences of food insecurity, as demonstrated by this pantry representative’s wide ranging description of what a pantry can offer:

*once people come and realise that you are there and you can*,* they can talk to you … then they come for food but also to ask for support and help to resolve abuse*,* issues with you know housing tenancy*,* child issues you know*,* like*,* parenting and you know resolving issues of the school. You know whatever**** CFP representative 4***.

Further, some of the additionality offered by CFPs, such as cooking and budgeting classes, appeared, to some participants, as reinforcing a belief that people experience food insecurity because of an inherent lack of skills and knowledge rather than a lack of income:

*you’ll see like a poster*,* and it will be like ‘learn how to cook on a budget’… you’re assuming that they don’t know how to cook on a budget*,* you’re basically saying the reason you’re skint is because you’re not cooking on a budget. Whereas we know the reason people are skint is because they’re not often getting what they’re entitled to*,* they’re often not entitled to enough**** Community food development worker***.

### Challenges of addressing food insecurity at community level

There were a number of challenges identified by stakeholders and within policy accounts related to CFPs’ role in tackling food insecurity. Whilst cross-sectoral collaboration and partnership working were considered key to responding to food insecurity, this was difficult to achieve in practice, and particularly in the context of the CFP model, which faced issues related to territorialism, food supply chains and, more broadly, fundamental limitations of tackling food insecurity solely via community level interventions. These challenges will now be discussed in turn.

#### Partnerships & territorialism

At the community level, multi-sectoral partnerships, operating between different levels of decision-making, were often described as crucial in tackling food insecurity, as was building robust relationships between local organisations.

*partnerships at a local level are really*,* really key**** Food bank representative 2***.

Despite the clear importance placed on relationships, there appeared to be consistent tensions between different projects, in particular between food banks and CFPs, and territorialism appeared to exist. For one interviewee, this was directly attributed to a competitive and unstable funding environment, something which over half the research participants cited as an ongoing challenge for community level projects:

*we had the working group for … food insecurity and it was extremely ugly to watch that unfolding*,* you know amongst the various community groups and agencies and charities and organizations. Going wild… to kind of secure funding … it was a power grab kind of thing going on there. Crazy crazy power grab.**** CFP representative 4***.

Whilst for others, territorialism was driven by the different long-term objectives of organisations, in particular projects who wish to expand (i.e., CFPs) vs. projects who, at a policy level, are earmarked for closure (i.e., food banks). This tension was described by a food bank representative who expressed concerns around these potentially contradictory messages coming from pantries and foodbanks around their longer-term ambitions:

*I think it’s been interesting to see these relationships develop and think about how we might need to work together…. And how important that’s going to be as more as more pantries open up*,* possibly or as they expand those that are already there*,* it’s just making sure that hopefully we’re all speaking with the same voice when it comes to the wider issues**** Food bank representative 1***.

Indeed, despite CFPs and food banks often working in the same space, responding to the same issues, it was rare for representatives to describe any partnership working taking place *between* these types of projects.

#### Flawed supply chains

Another key challenge for CFPs was food supply. The possibility of choice, seen as so integral to the pantry model, appeared to be constrained by both the supply of food and the demand on the project, with participants talking at length around issues with ensuring the pantries are well stocked and able to meet the needs of their members:

*So*,* the biggest challenge is the food… we have bimonthly meetings with the pantries*,* and that is by far the biggest issue*,* the biggest challenge that comes up.**** CFP representative 2***.

For some, this highlighted an inherent flaw in the CFP model: an over reliance on surplus or waste food, which is not sufficient for the level of expansion and does not necessarily provide quality or choice for their members:

*there just isn’t the right supply chain for that system to be rolled out to every community… that’s a logistical barrier … that I think people are overlooking*,* they think there’s an endless amount of food waste to be redistributed to people who can’t afford food and it’s just not the case or it’s not the right food and it’s not the food that people would choose**** Food policy representative 1***.

This concern about supply infrastructure for CFPs was also explicitly referenced in Scottish Government’s Food Insecurity Fact Sheet, where new food-based projects were required to demonstrate how issues related to food supply, in terms of both quantity *and* quality, will be mitigated or removed [36].

#### Community level approaches

In addition to these operational concerns, some participants were also critical of a community-level approach in a general sense as the preferred means of addressing food insecurity. They reflected on the need for higher-level action that addressed the root causes of food insecurity (and health inequalities) through social policy:

*Like first and foremost*,* families should be able to put food on their own tables. Do you know what I mean? We shouldn’t have to intervene with this kind of project… So first and foremost*,* I think there’s a policy shift required. …. it’s really bad. It’s just what what they’re doing to people’s mental health is just appalling. You know aside from what they’re doing economically to people*,* it’s just… really turning the screw*,* isn’t it? It’s so bad.**** ’ CFP representative 3***.

Indeed, resolving food insecurity was routinely described as fundamentally complex, requiring input, action and collaboration from multiple stakeholders at multiple levels of decision-making. This complexity was also reflected strongly in the policy documentation which located tackling food insecurity within a much broader policy and legislative framework spanning across multiple domains of action including gender inequality, fair work, child poverty, human rights and social security [35].

### Articulations of health inequalities in relation to food insecurity & CFPs

Research participants provided complex narratives and perceptions of health, often detailing nuanced understandings of the social determinants of health and the way that these intersected with food insecurity. However, this nuanced perspective was not so evident in the policy documents, which tended to provide relatively limited accounts of health in relation to food insecurity, often focussing on ‘healthy eating’ and / or social isolation and loneliness rather than reflecting the full spectrum of consequences for health which can occur as a result of experiencing food insecurity. Psychosocial harms, in particular related to dignity and choice, featured heavily in interviewee conceptualisations of health, with some disagreement on whether CFPs were able to fully mitigate the potential harms which arise from experiencing food insecurity. These points are detailed below.

#### Varied narratives of the relationship between CFPs & health

Perceptions of health varied across and within accounts. Whilst strong themes of individualisation [40] emerged, i.e., where participants described a lack of personal skills or knowledge as a driver of food insecurity, these themes were seldom presented as the only factor at play and were often located within a much more complex narrative of the social determinants of food insecurity and health. Indeed, every participant acknowledged the socio-economic determinants, particularly those related to income and poverty, of food insecurity, and in some cases provided nuanced descriptions of how this related to health, for example the description provided by a community food development worker of the evident impacts of food insecurity which they witness in their role:

*It’s the daily stress of insecurity*,* like whether it’s food*,* housing*,* employment*,* whatever and and what that does to folks’ mental health as well as like physical is just so so so visible*. ***Community food development worker***.

Others discussed how food insecurity, diet and health are interlinked at an individual level, revealing multi-layered perceptions of both food insecurity and health:

*Of course*,* there is a major overlap between food insecurity and dietary ill health. And in terms of what food you’re able to access*,* how often*,* the quality and the quantity and all of these kinds of things*,* but also just people have a really high coincidence of having a health condition and being food insecure. Then they cycle back and forth between each other**** Food policy representative 1***.

What is less clear is the role which pantries play in tackling or mitigating inequalities in health, when asked to consider this, participant accounts tended to narrow somewhat on either nutritional health and wellbeing, social isolation or physical fitness.

*So*,* the pantry again is about*,* you know breaking down those barriers… so if they’re socially isolated they can work with people*,* they can*,* they can work on their allotment…. They get that sense of satisfaction. … and doing walking groups and stuff as well. So absolutely health and wellbeing is a huge part of this as well’**** CFP representative 2***.

This narrowing was also evident in the policy documents which rarely mentioned health, and if they did tended to focus very broadly on nutritional wellbeing, for example referencing the importance of ‘healthy eating’ [34] or focusing on specific mental health problems related to social isolation and loneliness, whilst largely ignoring the wider complexities related to the social determinants of health. Interestingly, in the context of nutritional health and well-being, the *quality* and *variety* of food on offer was rarely discussed by participants who tended to focus largely on the quantity available. For one participant this highlighted the absence of a ‘health’ lens being applied, which, if present may have changed the developed models entirely:

*what is often missed is this health question. And I think if you’ve stuck health right into the starting point of some of the community pantry conversations*,* we would have developed a very different set of solutions**** Food policy representative 1***.

#### A return to dignity and choice

One pathway which was universally acknowledged by participants was the psychosocial harm that can be inflicted through the experience of food insecurity, with beneficiaries of services regularly described as ‘mortified’, ‘ashamed’ or ‘embarrassed’. It is here that the concept of choice appears to become integral to the role of CFPs once more. The ability to choose food for yourself was seen by some as an effective remedy for the perceived indignity of having to access charitable provisions in order to feed yourself and/ or your family, as detailed in this pantry representative’s perception of how this affected someone accessing support:

*So*,* you know witnessing this woman coming in like you know*,* the whole physical demeanour you know the body*,* the body language… I’m so sorry to be here. Could I have something and then say oh you can just help yourself …next time that she came in*,* she was an erect*,* dignified person**** CFP representative 4***.

However, the value placed on this conceptualisation of choice was critiqued by others, whose accounts of choice extended much further to whether people have the financial freedom to choose *where* they buy food. In this context, the CFP model was seen to be lacking and unable to provide the level of autonomy needed to resolve the impact of food insecurity, calling into question whether the CFP model can ever fully mitigate the related psychosocial harms:

*the point about choice is that people need to be able to choose whether they go to places like this… rather than having to do so because they haven’t got enough money**** Food bank representative 2***.

In short, the conceptualisations of the pathways through which community-level interventions, such as the pantries, interact with health inequalities were multi-layered and complicated. The social determinants of food insecurity and health were widely recognised by participants although there was a notable silence in the policy documentation, which did not appear to acknowledge the interlinked and complex pathways through which food and health intersect. Similarly, the nutritional value and quality of the food on offer was often absent from conversations around the role of CFPs, with relatively narrow or unclear conceptualisations of their potential and realised health impacts. There were, however, strong narratives which placed the ability to choose food items as integral to tackling the psychosocial harms of food insecurity, but these conceptualisations were not without critics, who understood *choice* and *dignity* in terms of financial autonomy and the freedom to choose *where* to shop, not just which items to choose from a food charity.

## Discussion

This paper explored stakeholder and policy views on CFPs, with a particular focus on health and its social determinants. Our document analysis showed Scotland’s policy commitment to transition away from emergency food aid as the primary response to food insecurity. However, findings suggest that the CFP model, whilst celebrated by some, shares some of the same issues faced by food banks when tackling food insecurity at a community level. Indeed, it appears that tackling food insecurity, and health inequalities, solely on this level, is challenging, if not impossible.

Despite consensus that food insecurity requires cross-sectoral and multi-level responses, there appear to be significant issues with territorialism in the delivery of CFPs. These issues relate to a competitive funding environment, contradictory long-term objectives and fundamental issues with supply chains that are predominantly reliant on waste food. In addition, in keeping with long standing debate on interventions to tackle health inequalities [35], strong critiques emerge around the capacity of community-level projects to tackle the upstream, income-based, drivers of food insecurity, which are not within their reach or remit to address.

This study showed that one conceptualisation of CFPs, for some denoting a distinguishing feature from food banks, was the link to a range of services to support people with broader aspects of their lives beyond food. However, what these services were, or how this would play out in practice, or in fact what issues these services would solve, were less clear. Some mentioned a detached and individualistic response, related to the need to increase knowledge and connections, while others talked about the need to provide services to support income maximisation. For a functioning referral system between services, there requires to be some degree of collaboration between organisations and services. However, given the funding pressures and territorialism highlighted in our study, this is likely challenging to achieve in practice. Whilst there is long standing scepticism about the capacity of food based projects to reduce food insecurity in any significant way [41], one study has evidenced a small reduction in experiences of *acute* food insecurity amongst service users who accessed food support in a community hub setting [42]. Further work to understand how CFPs connect to wider services, including both the route to access as well as the type of service and outcome would enable much better understanding of the role of CFPs in response to food insecurity and health inequalities more generally.

The concept of choice was also regarded as integral to the CFP model, something routinely intertwined with notions of dignity in interview and policy accounts. However, pervasive and well-known issues with food supply appear to undermine CFPs’ ability to fully meet the dietary needs and choices of people who access them. The fact that much of the food is sourced from waste or surplus redistribution food chains is also not without concern. Studies have found that people who access food in this way can feel shame as a result of being given food which is past its expiration date [19], with repeated concerns raised in Scotland about the quality of the food provided via surplus redistribution, which can be both nutritionally limited and inappropriate [43]. Similarly, in keeping with some perspectives in this study, theorists have expressed deep concern about the *expansion* of surplus food redistribution [3, 25], suggesting it serves only to ‘contain’ the issue of food insecurity and that its expansion largely benefits the interests of ‘Big Food’, i.e., supermarkets, who are able to redistribute surplus or waste food rather than embark on any systems change which would prevent such surplus or waste food from existing in the first place [3]. Indeed, some theorists claim that ‘[surplus food redistribution] paradoxically reinforces the same problems it attempts to solve’ [25], suggesting any attempts to roll out a model which is wholly reliant on these supply chains requires urgent critique.

Alongside this, despite claims that CFPs are ‘more dignified’, concepts such as ‘dignity’ or ‘stigma’ are not always easily measured [44, 45]. Investigation of how these models are experienced might shed light on whether the level of choice and mode of access does indeed mitigate or remove the psychosocial harms of food insecurity in the significant ways suggested by some interviewees. It is also unclear how or if these novel approaches remove or mitigate other detrimental health impacts associated with food bank use and food insecurity more generally. These include both direct and indirect impacts such as stress and worry [12], the inability to adhere to speciality diets [14], along with limited opening times, and in some cases, what appears to be policed access [9]. Indeed, accounts included in this study simultaneously suggest CFPs interact with food insecurity in multiple ways via *addressing* the issue [s] (through multiple social interventions, including income maximisation), *mitigating* (through food support) and potentially *exacerbating* social inequalities (through inadequate provision of above services and mitigations) and the health differences that flow from these. Academics experts have warned of the dangers associated with simply seeking to ‘build better food banks’ [9] rather than investigate and explore the ways that income-based drivers of food insecurity might be adequately tackled [10, 41]. However, given the intense expansion of the food aid landscape in recent years [3], and in light of this study, it appears there is work to be done to understand and interrogate the wider health impact of CFPs, and similar models, as well as investigate any potential costs or benefits for the people who access them.

### Strengths and limitations

Although this study provides a helpful snapshot of frontline and policy conceptualisations and perspectives of CFPs, the pool of interviewees was small, a limitation it is important to recognise. To remedy this, we have triangulated our data through our policy analysis and rooted the findings in wider literature related to food insecurity in the UK and beyond. We believe this study contributes to ongoing and urgent conversations about novel, community-based responses to food insecurity and reveals some pertinent questions and future areas for investigating how CFPs operate and are experienced. This includes the need to understand in more detail how CFPs operate within the system of support available for people experiencing food insecurity, specifically exploring how they connect to other services and are experienced by the people who access them. In addition, this study has highlighted the need to critically appraise the design of CFPs, particularly in relation to their food supply chains and how they interact with other food-based responses, such as food banks.

## Conclusion

Whilst this study has identified some further areas of research to fully understand the role of CFPs, it also adds to a body of literature which recognises that community-level interventions are fundamentally constrained when tackling the social determinants of food insecurity and health [3, 9, 41, 46, 47]. Indeed, until we have a better understanding of their potential and realised impacts, both positive and negative, expansion of CFPs should be viewed with caution, and in the same vein as traditional emergency food provision: a symptom of, rather than a solution to, the problem of food insecurity.

## Electronic supplementary material

Below is the link to the electronic supplementary material.

Supplementary Material 1: Additional file 1 - (Topic Guide for interviews): This document details the topic guide developed for all of the interviews with stakeholders, including key areas and questions covered in each

## Data Availability

Raw data generated and analysed in this study are not publicly available. This is due to the conditions on which consent was obtained and to protect participant confidentiality. Data requests can be made to Anna Baillie, a.baillie.1@research.gla.ac.uk.
