# Supplementary material for: An exploration of community food pantries in Scotland: strategic and operational perspectives on addressing food insecurity and health inequalities
Source: BMC Public Health. 2024 Oct 18;24:2881. doi: 10.1186/s12889-024-20421-z (PMC11490137; doi:10.1186/s12889-024-20421-z)
Supplement: Supplementary file 1 — Supplementary Material 1: Additional file 1 - (Topic Guide for interviews): This document details the topic guide developed for all of the interviews with stakeholders, including key areas and questions covered in each [file 12889_2024_20421_MOESM1_ESM.docx]

# **Stakeholder views on community-led interventions to address food insecurity**

## Topic guide

### **Introductory section**

- First of all, thank you so much for agreeing to talk to me and for taking part in this research.
- My name is xx, and I’m a research assistant at the University of Glasgow, and part of a team working on this project, which aims to explore expert stakeholder views on the opportunities and challenges of community-level initiatives to address poverty, and specifically food insecurity in Scotland. We are particularly interested in exploring the community pantry model, and its potential for addressing food insecurity. The findings from this study will hopefully inform future studies to support community-driven change, and future policy to tackle food insecurity. We are so grateful that you have agreed to share your knowledge and experiences with us.
- [Reiterate key points from consent form, check verbally that participant has read the information sheet and is happy to participate and be recorded]
- Thank you for sending over your consent form.
- Before starting, I just want to highlight and check a few things. I just want to check that you had a chance to look over the information sheet and privacy notice and are happy to take part.
- All information collected during the study will be treated in accordance with legal and ethical requirements for data handling and storage. Although we may use information you provide in publications and reports (for example excerpts of what you say in quotations), we will not use information that could identify you or your organisation. We realise that within particular areas/professional communities, it may be possible for others to identify you from your experiences, as such we will take extra care to anonymise quotations as appropriate to avoid accidental disclosure.
- Your taking part is voluntary, and you are free to withdraw at any time without given a reason.
- Do you have any questions?
  - [Verbal confirmation of consent]: Do you consent to take part in this research, and do you give consent for me to record this interview?
- Before starting, I’d just like to highlight that there is absolutely no right or wrong answers to these questions, just a chat really. If there is any you would rather not answer or are not sure about, that is totally fine.

**Introductory questions:**

So just to start off with a couple of introductory questions, just generally about food insecurity, and also your professional role. So firstly…

- What does food insecurity mean to you?
- Can you tell me a little bit more about your professional role, and how it relates to food insecurity?
  - Specific role, department/organisation
- What are the goals of the organisation you work for?
  - Where does your organisations goals come from?
  - What does your organisation do around tackling health inequalities?
  - How do you judge your own success?
- Other organisations
  - How do you work with other organisations to achieve your goals?
  - Thinking about the organisation you work for, do you think their goals are similar/different to other organisations working in this area? How so?

### **Focus on community pantry model:**

It would be good now to talk a little bit more about community pantries in particular.

- Can you talk about your awareness of and experiences relating to community pantries?
  - Can you describe the community pantry model that you are familiar with?
  - Why and when do you think people use community pantries? Who do you think uses them? Who are they meant for and who comes through the door?
  - How do you measure the impact/success of community pantries?
- How do community pantries work to alleviate food insecurity?
- Where do community pantries fit in the wider food insecurity response?
  - How do community pantries compare to other responses to food insecurity such as a food banks or cash-based responses?
- Are there other benefits to the community, positive impacts beyond easing food insecurity? (prompts: *community cohesion; social support; cross-service integration*)
  - I’m wondering if you could talk a bit about where health might come into this/what might be the potential impacts on health?
  - How might community pantries improve health and wellbeing?
- Are there any unintended negative impacts of community pantries? Or issues you are aware of? (prompts: *impact on individuals, businesses, communities)*
- Do you think there is potential in rolling out community pantries to address food insecurity more widely? (prompts: *facilitators/barriers to this)*
  - Do you think scale-up of community pantries would be supported by food manufacturers and retailers?
  - What potential issues might there be with corporate involvement in tackling food insecurity?
  - Other than redistributing their waste food, what other roles might supermarkets play in reducing levels of food insecurity?
- Are you aware of the community pantry network?

### **Focus on community-level /context:**

Now thinking more generally about the community context…

- What does something being described as a ‘community initiative’ mean to you?
- What does it mean to have a community led initiative? How do you make sure the community are involved?
- How important do you think it is that interventions/initiatives are driven by the community?
  - How do you maintain community input to these initiatives?/difficult to keep the community-led nature?
- What are the barriers and facilitators to delivering the aims of community-level initiatives to address food insecurity in the community?
- Are there any other influences beyond the scope of what we have discussed so far? (prompts: *policy; economic influences)*
- *How do you think health is usually framed within community-level interventions?*
- *Any examples of really positive community-led approaches to improving health and wellbeing?*

### **Focus on partnership working:**

Thinking more broadly about partnership working…

- Can you describe the key agencies/partners that you work with who have a role in responding to food insecurity? (prompts: *local level up to government level)*
- Are there any particular successful partnership working models that you have been involved in?
- Are there any specific challenges you have encountered around working in partnership to address food insecurity?

### **Future interventions/next steps**

Lastly, thinking about the future…

- What do you see as the next steps to addressing food insecurity? (prompts: *feasibility? Challenges?)*
  - What would you like to change regarding the way food insecurity is currently being dealt with?
- What actions do you think should be prioritised in working to address food insecurity?
  - In what ways might your priorities differ from others working to address food insecurity? e.g. third sector; commercial; policy
- What difference might it make to a population in terms of health?
- Is there anything important that researchers and policymakers need to consider when exploring options for tackling food insecurity?
- Are there any other approaches for tackling food insecurity that we haven’t discussed yet?

If hasn’t come up

- What has been the impact of the COVID pandemic on both food insecurity itself, as well as actions/initiatives to address food insecurity?

### **Close interview**

- Is there anything else you would like to add, that we haven’t already talked about?
- [Interviewee suggestions] Is there anyone else you recommend that we talk to?
- Thank you!
